# SNHG15 is a negative regulator of inflammation by mediating TRAF2 ubiquitination in stroke-induced immunosuppression

**DOI:** 10.1186/s12974-021-02372-z

**Published:** 2022-01-03

**Authors:** Huiling Sun, Shuo Li, Zhaohan Xu, Chengfang Liu, Pengyu Gong, Qiwen Deng, Fuling Yan

**Affiliations:** 1 General Clinical Research Center, Nanjing First Hospital, Nanjing Medical University, Nanjing, 210006 People’s Republic of China; 2 Department of Neurology, Affiliated ZhongDa Hospital, School of Medicine, Southeast University, No. 87 Dingjiaqiao Road, Nanjing, 210009 People’s Republic of China; 3 Department of Neurology, Nanjing First Hospital, Nanjing Medical University, No. 68 Changle Road, Nanjing, 210006 People’s Republic of China

**Keywords:** Long noncoding RNA, Ischemic stroke, SNHG15, Immunosuppression, TRAF2

## Abstract

**Background:**

Abnormal expression of long noncoding RNAs (lncRNAs) has been reported in the acute stage of acute ischemic stroke (AIS). This study aimed to explore differential lncRNA expression in the subpopulations of peripheral blood mononuclear cells (PBMCs) from AIS patients and further evaluate its underlying mechanisms in stroke-induced immunosuppression.

**Methods:**

We reanalyzed lncRNA microarray data and investigated abnormally expressed lncRNAs in the subpopulations of PBMCs by magnetic cell sorting and real-time quantitative PCR. The potential mechanism of small nucleolar RNA host gene 15 (SNHG15) was explored through in vitro and in vivo approaches.

**Results:**

The stroke-induced SNHG15 acted as a checkpoint to inhibit peripheral inflammatory responses. Functional studies showed that SNHG15 promoted M2 macrophage polarization. Mechanistically, SNHG15 expression was dysregulated through the Janus kinase (JAK)-signal transducer and activator of transcription 6 (STAT6) signaling pathway. SNHG15, localized in the cytoplasm, interfered with K63-linked ubiquitination of tumor necrosis factor receptor-associated factor 2 and thereby repressed the activation of mitogen-activated protein kinase and nuclear factor kappa-B signaling pathways and prevented the production of proinflammatory cytokines. Administration of an adenovirus targeting SNHG15 improved stroke-induced immunosuppression in mice.

**Conclusions:**

This study identified SNHG15 as a negative regulator of inflammation in stroke-induced immunosuppression, suggesting it as a novel biomarker and therapeutic target in stroke-associated infection.

*Trial registration* ClinicalTrials.gov NCT04175691. Registered November 25, 2019, https://www.clinicaltrials.gov/ct2/show/NCT04175691.

**Supplementary Information:**

The online version contains supplementary material available at 10.1186/s12974-021-02372-z.

## Background

Stroke is a major cause of long-term disability and death in adults worldwide [1]. Stroke-associated infection (SAI) is one of the common complications after stroke occurring especially in severe stroke, and it prolongs hospital stays and augments mortality rates [2]. In view of the clinical significance of SAI, more investigators have focused on early prediction and precise prevention of SAI including the acute ischemic stroke-associated pneumonia score, prophylactic antibiotic therapy and active airway management [3–5]. However, such methods are unavailable to decrease the incidence of SAI and improve their prognoses [4, 5].

Accumulating evidence from clinical and experimental studies demonstrates that stroke-induced immunosuppression (SIIS) makes patients more susceptible to post-stroke infections [6–9]. SIIS is characterized by an imbalance in the systemic cellular immune response, including dysfunction of monocytes and a rapid decrease in peripheral lymphocyte subpopulations, both of which increase the risk of SAI [10]. Furthermore, SIIS is an independent predictor for SAI, suggesting that immuno-inflammatory response plays an important role after stroke [6, 11]. Our previous study found that the sympathetic pathway is enhanced after ischemic stroke and subsequently induces significant changes in the levels of cytokines in the periphery [9]. However, the pathogenesis of SIIS remains elusive.

Long noncoding RNAs (lncRNAs) are a set of non-protein-coding transcripts greater than 200 bases in length [12], and are involved in a series of physiological and pathological processes, including cell differentiation, embryonic development and regulation of the cell cycle [13]. A number of lncRNAs have been reported to have diagnostic and therapeutic values in ischemic stroke [14–16]. Our previous study showed differential expression of lncRNAs in peripheral blood mononuclear cells (PBMCs) in acute ischemic stroke, revealing that a panel of altered lncRNAs (linc-DHFRL1-4, SNHG15, and linc-FAM98A-3) may serve as a novel diagnostic tool [17]. Nevertheless, whether these lncRNAs are involved in SIIS is unknown.

In this study, we investigated differential expression profiles of these lncRNAs in the subpopulations of PBMCs after acute ischemic stroke (AIS) and proposed a model in which interleukin-4 (IL-4) induces upregulation of SNHG15, which serves as a repressor of the inflammatory response through inhibiting K63-linked ubiquitination of tumor necrosis factor (TNF) receptor-associated factor 2 (TRAF2).

## Methods

### Study population

All blood samples in this study were obtained from participants in an observational study of circulating non-coding RNAs in acute ischemic stroke (AISRNA) (www.clinicaltrials.gov, NCT04175691), which was a multiset, hospital-based, case–control study for the detection of noncoding RNA expression in AIS. LncRNA microarray profiling data were obtained from our previous study [17]. One hundred and seventy-three AIS patients were enrolled within 48 h after onset of stroke, which was confirmed by a high-density lesion on diffusion weighted imaging (DWI) of magnetic resonance (MR) or a new low-density lesion on a brain computed tomography scan. We excluded individual participants with a history of infectious disease within the 2 previous weeks, immune diseases, hemorrhagic infarction, and progressive malignancy. A total of 116 participants who were matched with the AIS patients for age, sex and medical history were enrolled as healthy controls (HCs) in the physical examination center. All participants offered informed consent. The study protocol was approved by the Ethics Committee of Nanjing Medical University (No. (2019)695) and Southeast University (No. 2018ZDKYSB193), and followed the tenets of the Declaration of Helsinki.

### Blood sampling and processing

Human blood samples (4–5 ml) were collected in a K2-EDTA containing tube (Vacutainer, BD, USA) within 48 h after onset of stroke. PBMCs were subsequently isolated within 2 h as previously reported [17]. Plasma was extracted from individual participants into a procoagulant tube (Vacutainer, BD, USA) for measurement of cytokine protein levels. Mouse blood samples were obtained from the retro-orbital plexus by enucleation to rupture the ophthalmic artery. Peripheral blood samples (1–1.5 ml) were collected in a K2-EDTA containing tube (Vacutainer, BD, USA) and processed within 2 h. After centrifugation at 1500 rpm for 10 min to remove plasma, blood samples were subjected to Ficoll density centrifugation (TBD Science, Tianjin, China) to isolate PBMCs according the manufacturer’s instructions.

### RNA isolation and quantitative real-time PCR (qRT-PCR)

RNA isolation and qRT-PCR were performed according our previous study [17]. Briefly, total RNA was extracted from cells (human and mouse) and brain tissues of mice with TRIzol reagent (Invitrogen, CA, U.S.A.) following the manufacturer’s protocol. The purity and concentration of RNAs were determined by a NanoDrop spectrophotometer (Thermo Scientific, MA, USA). Subsequently, RNA was reverse transcribed using a PrimeScript RT Reagent Kit (Takara, Dalian, China) and quantified using SYBR Premix Ex TaqTM II Kit (Takara, Dalian, China). Analysis was performed using GAPDH as the internal control. Expression levels of candidate RNAs were calculated with ΔCT or 2^−ΔΔCt^ values if necessary. Primers for qRT-PCR were synthesized by GENEray (Generay Biotech Co, Shanghai, China) and are listed in Additional file 1: Table S1.

### Cytokine protein analysis

Plasma samples from a total of 173 AIS patients and 116 corresponding HCs were used for measurement of cytokine protein levels. The concentrations of IL-4, IL-10, IL-6 and tumor necrosis factor α (TNF-α) in plasma were analyzed with a Navios flow cytometer (Beckman Coulter, California, USA). Mouse peripheral blood (1–1.5 ml) acquired from the retro-orbital plexus was collected in a K2-EDTA tube (Vacutainer, BD, USA) and centrifuged at 1500 rpm for 10 min. The plasma was separated and stored at a temperature of -80 °C when necessary, avoiding freeze–thaw cycles. The concentrations of the cytokines IL-4, IL-10, IL-6 and TNF-α in mouse plasma were measured with enzyme-linked immunosorbent assay (ELISA) Kits (Joyee Biotechnics Co. Ltd., Shanghai, China) according to the manufacturer’s protocol.

### Separation and purity of monocytes/macrophages

Human PBMCs were re-suspended for cell sorting. We selected CD14+ monocytes/macrophages by using CD14 MicroBeads and Isolation Kit as previously reported [18]. Unmarked lymphocytes were also collected after magnetic cell sorting. Subsequently, monocytes/macrophages were treated with a FITC-conjugated anti-human CD14 antibody (Biolegend, CA, USA). The purity of monocytes/macrophages was confirmed by flow cytometry (BD FACSCanton-II, BD Bioscience, Franklin Lakes, NJ, USA) following the manufacturer’s protocol. Data were analyzed with FlowJo 7.6 software.

### Cell culture

Human monocytes/macrophages were isolated after magnetic cell sorting and re-suspended in RPMI 1640 medium (Gibco) supplemented with 10% fetal bovine serum (Gibco). Human THP-1 monocytes, mouse RAW264.7 cells and HEK293T cells were obtained from the Cell Bank of the National Academy of Sciences (Shanghai, China) and incubated in RPMI 1640 medium supplemented with 10% fetal bovine serum for further study. THP-1 cells were treated with PMA (100 ng/ml, Sigma, Germany) in serum-free medium for 24 h to induce differentiation into macrophages.

### RNA FISH and protein immunofluorescence staining

The fluorescence in situ hybridization (FISH) array assay was performed using a Fluorescence In Situ Hybridization Kit (C10910; RiboBio, China) according to its protocol. The 5’ FAM-labeled SNHG15 probe was designed and synthesized by GenePharma (Suzhou, China). The probe sequences are shown in Additional file 1: Table S2. After incubation with the SNHG15 probe, cells were treated with a mouse monoclonal anti-CD14 antibody (Abcam, Cambridge, UK) or anti-TRAF2 antibody (Cell Signaling Technology, MA, USA) for 2 h at 37 °C and were then washed three times with PBS. These cells were incubated with a fluorescently labeled secondary antibody (Proteintech, Chicago, USA) for 1 h at 37 °C and washed three times prior to nuclear staining with DAPI for 10 min. Finally, the microscopy sides were mounted with anti-fade mounting medium (Abcam, Cambridge, UK).

### Nuclear and cytoplasmic isolation

Nuclear and cytoplasmic RNA was isolated from sorted monocytes/macrophages, THP-1 cells and RAW264.7 cells using a PARIS Kit (Invitrogen, USA) according to the manufacturer’s protocol.

### RNA pull-down and mass spectrometry

The biotin-labeled SNHG15 (sense and anti-sense) probe was purchased from RiboBio (Guangzhou, China). The SNHG15 probe was incubated with THP-1 cell lysates overnight at 4 °C, and complexes were then isolated with streptavidin magnetic beads (Invitrogen) for 1 h at 4 °C. The isolated complexes were processed by silver staining (Sangon Biotech, Shanghai, China) for mass spectrometry, and a specific band in the experimental lane was subsequently selected for further analysis. Additionally, the isolated complexes were analyzed by immunoblotting using an antibody specific for TRAF2 (Cell Signaling Technology, MA, USA).

### RNA immunoprecipitation and co-immunoprecipitation

RNA immunoprecipitation (RIP) and co-immunoprecipitation (Co-IP) assays were performed using an EZ Magna RIP Kit (Millipore, USA) according to the protocol. Briefly, cell lysates were incubated with RIP buffer containing magnetic beads that were preincubated with an anti-TRAF2 or anti-STAT6 (Cell Signaling Technology, MA, USA) antibody or IgG for 30 min at room temperature. The cell lysates were incubated with beads overnight at 4 °C. RNA/protein complexes were precipitated with protein G Dynabeads. Finally, the RNA or protein was purified and analyzed by qRT-PCR or western blotting.

### Ubiquitination assay

The expression plasmids HA-K48-UB (with all lysines except lysine 48 mutated to arginine) and HA-K63-UB (with all lysines except lysine 63 mutated to arginine) were purchased from Hanbio Technology (Shanghai, China). Ubiquitination assays were performed by immunoprecipitation and immunoblotting. Briefly, cell lysates were incubated with an anti-TRAF2 antibody (Cell Signaling Technology, MA, USA) overnight at 4 °C. Bound proteins were analyzed by immunoblotting with an antibody specific for ubiquitin (Invitrogen, MA, USA) after washing three times.

### Luciferase reporter assay

To confirm the association of signal transducer and activator of transcription 6 (STAT6) and the SNHG15 promoter, a pmirGLO reporter vector containing a constitutive or partially mutated promoter of SNHG15 was constructed. Monocytes were plated in 6-well plates and transiently cotransfected with the SNHG15 promoter or negative control using Lipofectamine 2000 (Invitrogen, MA, USA). Finally, luciferase activity was measured with a Dual-Luciferase Reporter Assay System (Promega, Wisconsin, USA).

### Statistical analysis

All statistical analyses were performed using GraphPad Prism 8.0 software (GraphPad Software Inc., CA, USA). Band intensities from the immunoblots were analyzed with ImageJ Software. Data are presented as the mean ± standard deviation (S.D.) values. Student’s t test (n = 2 groups) or analysis of variance (ANOVA) (n > 2 groups) was used to assess differences between groups. A p value less than 0.05 was considered statistically significant.

A description of methods, including construction of lentiviral and plasmid, western blot, the transient middle cerebral artery occlusion (tMCAO) model of mice, adenoviral vector construction and injection, neurological deficits evaluation, triphenyltetrazolium chloride (TTC) staining and infarct volume assessment, and PBMCs isolated from mice, are available in Additional file 1.

## Results

### Activation of inflammatory cytokines and differentially expressed lncRNAs after AIS

**Fig. 1:**
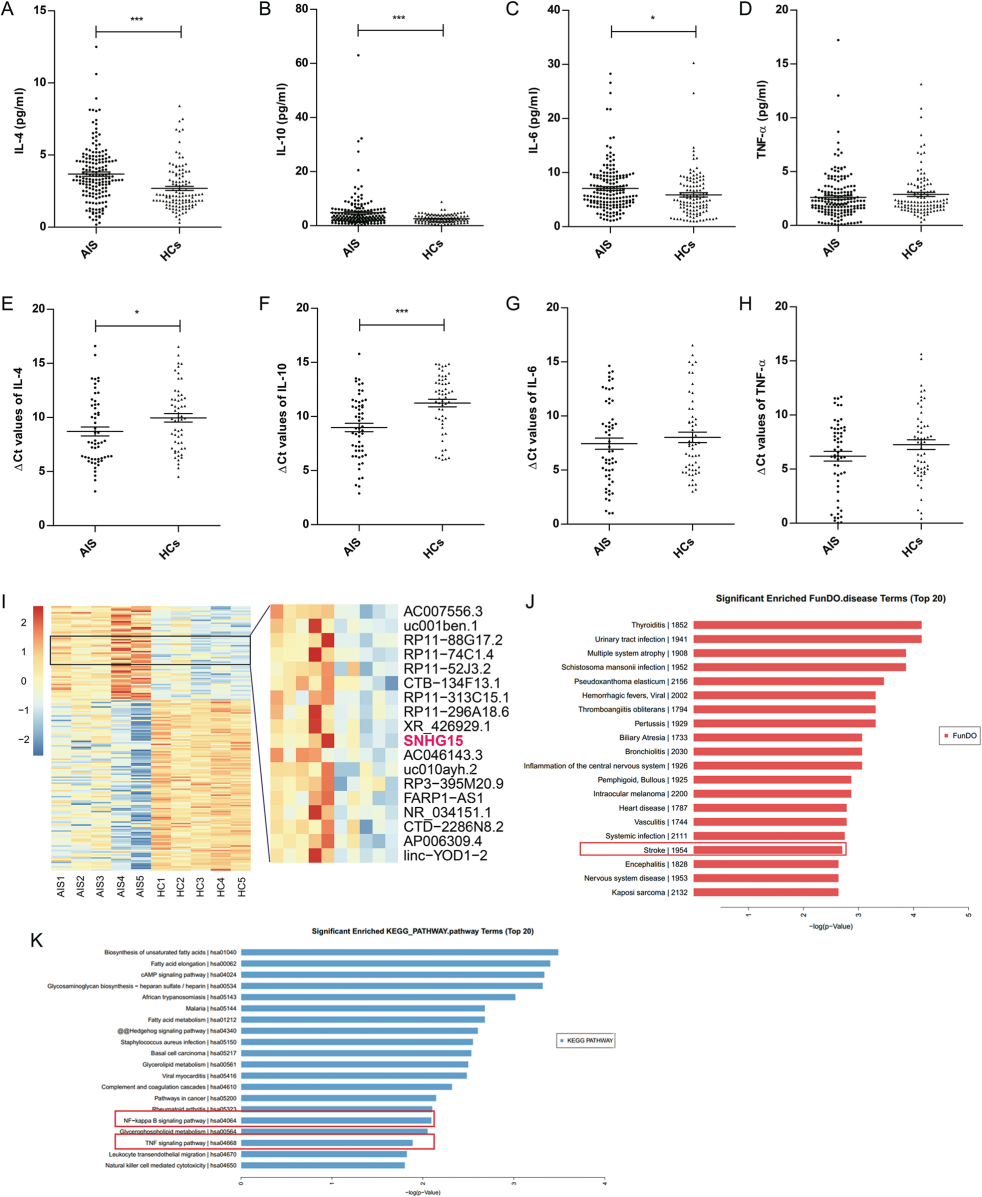
Differentially expressed cytokines and lncRNAs in AIS patients and HCs. Blood samples were collected within 48 h after onset of stroke and were subsequently isolated within 2 h. The levels of IL-4 (**A**), IL-10 (**B**), IL-6 (**C**), and TNF-α (**D**) in plasma were measured by a Navios flow cytometer. Expression of IL-4 (**E**), IL-10 (**F**), IL-6 (**G**), and TNF-α (**H**) was detected by qRT-PCR in PBMCs. **I** The heat map shows 70 upregulated and 128 downregulated lncRNAs in PBMCs identified by lncRNA microarray analysis. **J** Disease enrichment analysis showed the association of these lncRNAs with stroke. **K** Pathway enrichment analysis suggested the correlations of these lncRNAs with inflammatory pathways (NF-κB and TNF signaling pathways). * P < 0.05, *** P < 0.001

To identify the peripheral inflammatory response after AIS, we evaluated the changes in inflammatory cytokine levels in the plasma of 173 patients with AIS and 116 HCs. The protein levels of anti-inflammatory cytokines (IL-4 and IL-10) were obviously higher in AIS patients than in HCs (*P* < 0.001, Fig. 1A, B). The level of the proinflammatory cytokine IL-6 was slightly increased in patients with AIS (*P* < 0.05, Fig. 1C), but that of TNF-α was not significantly different (*P* > 0.05, Fig. 1D). qRT-PCR showed that IL-4 and IL-10 levels were significantly increased in the PBMCs of 56 AIS patients (*P* < 0.05, Fig. 1E and F), similar to the plasma level, but the levels of proinflammatory cytokines (IL-6 and TNF-α) were not changed (*P* > 0.05, Fig. 1G and H).

Our previous study showed that linc-DHFRL1-4, SNHG15, and linc-FAM98A-3 were substantially differentially expressed after AIS by lncRNA microarray analysis and qRT-PCR validation (Fig. 1I). Bioinformatics analysis showed that distinctive expression of lncRNAs was associated with stroke and various immune-associated genes (Fig. 1J and K).

### High expression of SNHG15 in monocytes/macrophages after AIS

**Fig. 2:**
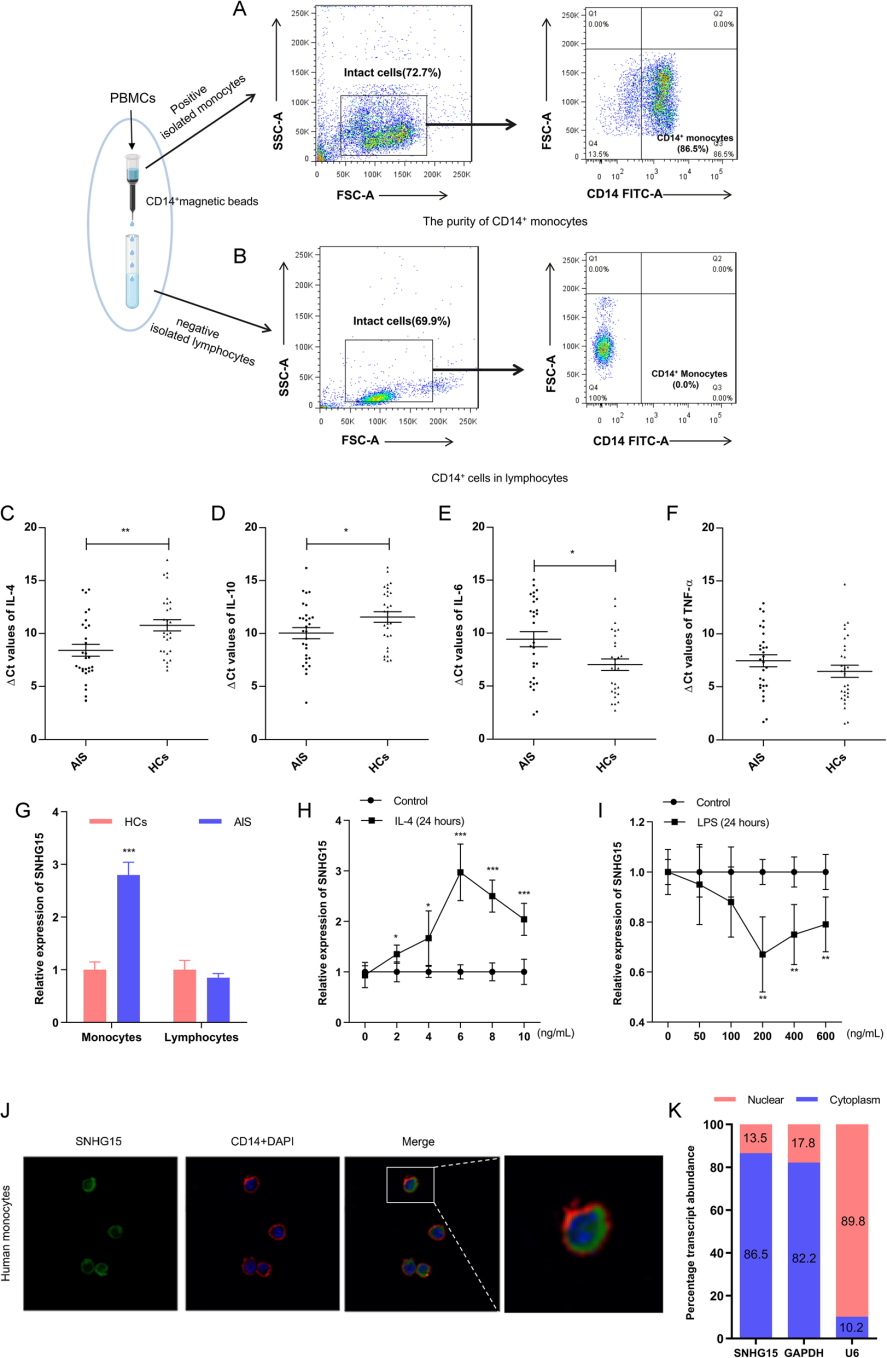
Validation of SNHG15 expression and localization. The proportions of positive isolated monocytes (**A**) and negative isolated lymphocytes (**B**) after sorting by magnetic beads were determined by flow cytometry. Expression of IL-4 (**C**), IL-10 (**D**), IL-6 (**E**), and TNF-α (**F**) in monocytes/macrophages isolated from PBMCs was detected by qRT-PCR and calculated with ΔCT. **G** SNHG15 relative expression in subpopulations of PBMCs. The expression of SNHG15 in IL-4-induced (**H**) or LPS-induced (**I**) monocytes isolated from PBMCs. **J** The colocalization of SNHG15 and CD14 + monocytes was assessed by FISH. **K** SNHG15 expression in the cytoplasm and nucleus of monocytes/macrophages. * P < 0.05, ** P < 0.01, *** P < 0.001

PBMCs primarily include monocytes/macrophages and lymphocytes. Thus, we further analyzed the expression profiles of lncRNAs in two different types of cells isolated from PBMCs by magnetic bead sorting. The sorted cells were confirmed by flow cytometry, and the results showed that monocytes accounted for 86.5% of these cells (Fig. 2A) and that the absence of monocytes was observed in the lymphocytes isolated by negative section (Fig. 2B). Subsequently, SNHG15 expression was detected in sorted monocytes and lymphocytes. SNHG15 expression in monocytes was higher than that in lymphocytes (*P* < 0.001, Fig. 2G), but linc-DHFRL1-4 and linc-FAM98A-3 expression remained unchanged (Additional file 1: Figure S1). Additionally, we measured the transcription levels of inflammatory cytokines in monocytes. Anti-inflammatory cytokines (IL-4 and IL-10) and proinflammatory cytokines (IL-6) were significantly increased in monocytes after AIS (Fig. 2C, 2D and 2E), but TNF-α was not (Fig. 2F). These results suggested that SNHG15 may be involved in stroke-induced immunosuppression.

### SNHG15 in monocytes/macrophages is induced by IL-4 and inhibited by LPS

**Fig. 3:**
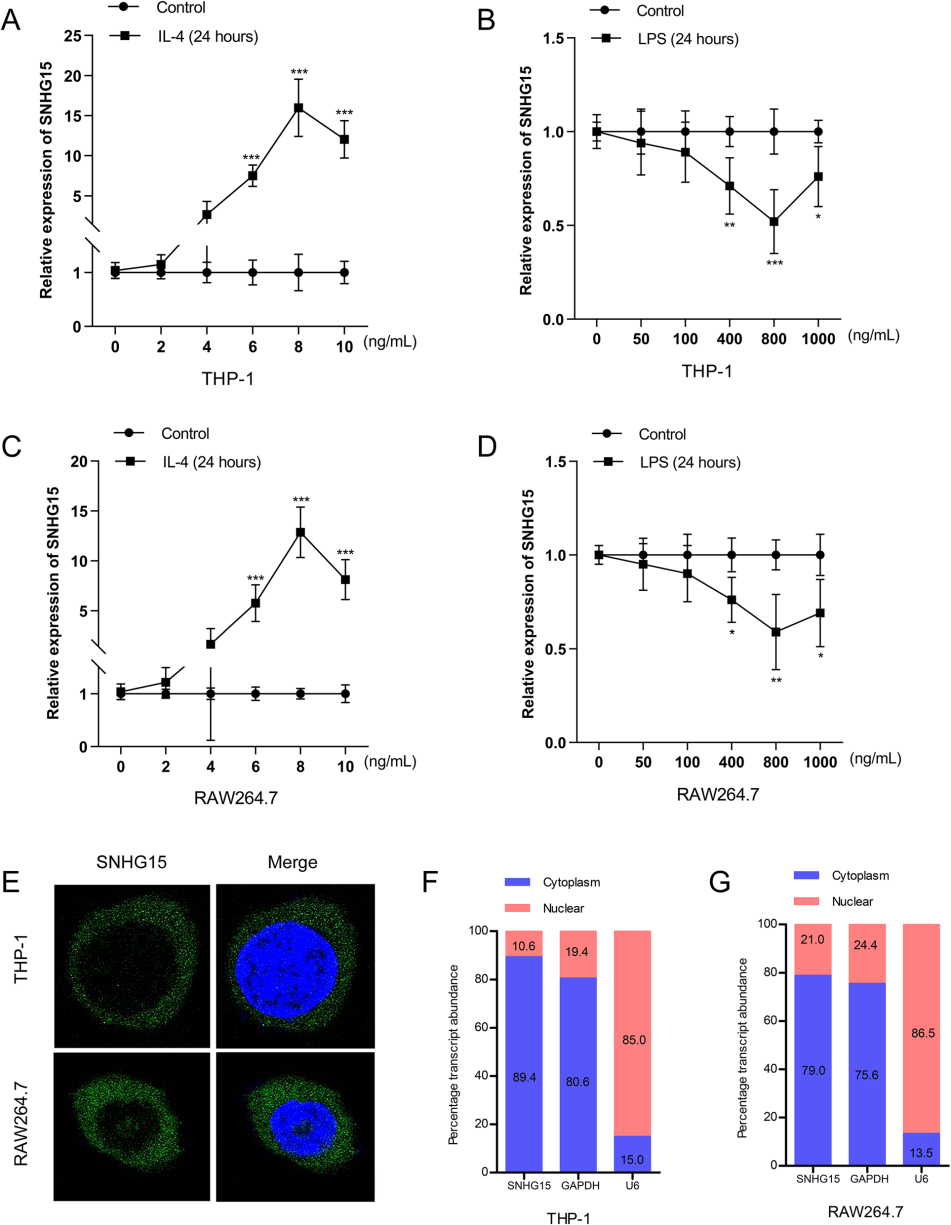
SNHG15 expression in macrophages stimulated by IL-4 and LPS. Expression of SNHG15 in THP-1 and RAW264.7 cells was induced by IL-4 and LPS (**A**–**D**). **E** The localization of SNHG15 in THP-1 and RAW264.7 cells was assessed by FISH. SNHG15 expression in the cytoplasm and nucleus of THP-1 (**F**) and RAW264.7 (**G**) cells was detected by qRT-PCR. * P < 0.05, ** P < 0.01, *** P < 0.001

To further explore the potential mechanism of SNHG15 in stroke-induced immunosuppression, we investigated the response of SNHG15 to IL-4 or lipopolysaccharide (LPS) in sorted monocytes/macrophages by qRT-PCR. SNHG15 expression in macrophages was induced by in a dose-dependent manner and peaked at an IL-4 concentration of 6 ng/mL (Fig. 2H). LPS significantly repressed SNHG15 expression at a concentration of 200 ng/mL (Fig. 2I). Similar results were observed in THP-1 and RAW264.7 cells (Fig. 3A–D), but linc-DHFRL1-4 and linc-FAM98A-3 expression was not changed (Additional file 1: Figure S2). As the function of a lncRNA depends on its subcellular localization, we observed that SNHG15 localized to the cytoplasm in sorted monocytes, THP-1 cells and RAW264.7 cells by FISH (Figs. 2J and 3E) and qRT-PCR (Figs. 2K and 3F, G).

### SNHG15 regulates macrophage polarization

**Fig. 4:**
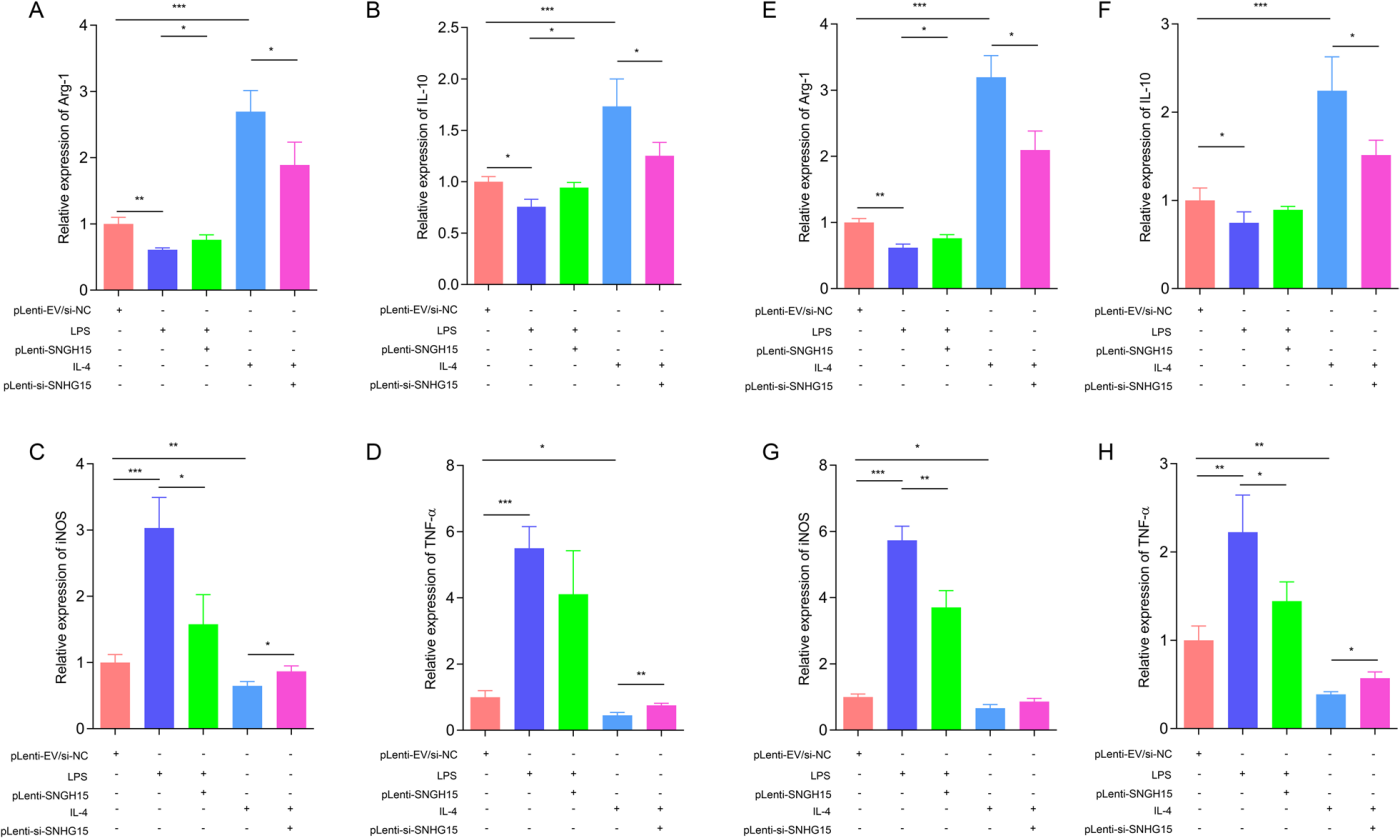
SNHG15 promotes M2 polarization of macrophages. (**A**–**D**) Effects of forced expression or silencing of SNHG15 on the expression of inflammatory factors induced by IL-4 or LPS in THP-1 cells. **E**–**H** Effects of forced expression or silencing of SNHG15 on the expression of inflammatory factors induced by IL-4 or LPS in RAW264.7 cells. * P < 0.05, ** P < 0.01, *** P < 0.001

To confirm the functional role of SNHG15 in the IL-4- or LPS-induced inflammatory response in monocytes/macrophages, we constructed lentiviral vectors to promote or suppress SNHG15 expression in THP-1 and RAW264.7 cells (Additional file 1: Figure S3). In LPS-mediated macrophages differentiated from THP-1 cells, overexpression of SNHG15 promoted the expression of Arg-1 and IL-10 but inhibited inducible nitric oxide synthase (iNOS) and TNF-α expression (Fig. 4A–D). The opposite results were found in IL-4-activated macrophages differentiated from THP-1 cells (Fig. 4A–D). Similar results were observed in RAW264.7 cells (Fig. 4E–H). These findings suggested that SNHG15 contributes to M2 macrophage polarization.

### SNHG15 interferes with K63-linked ubiquitination of TRAF2

**Fig. 5:**
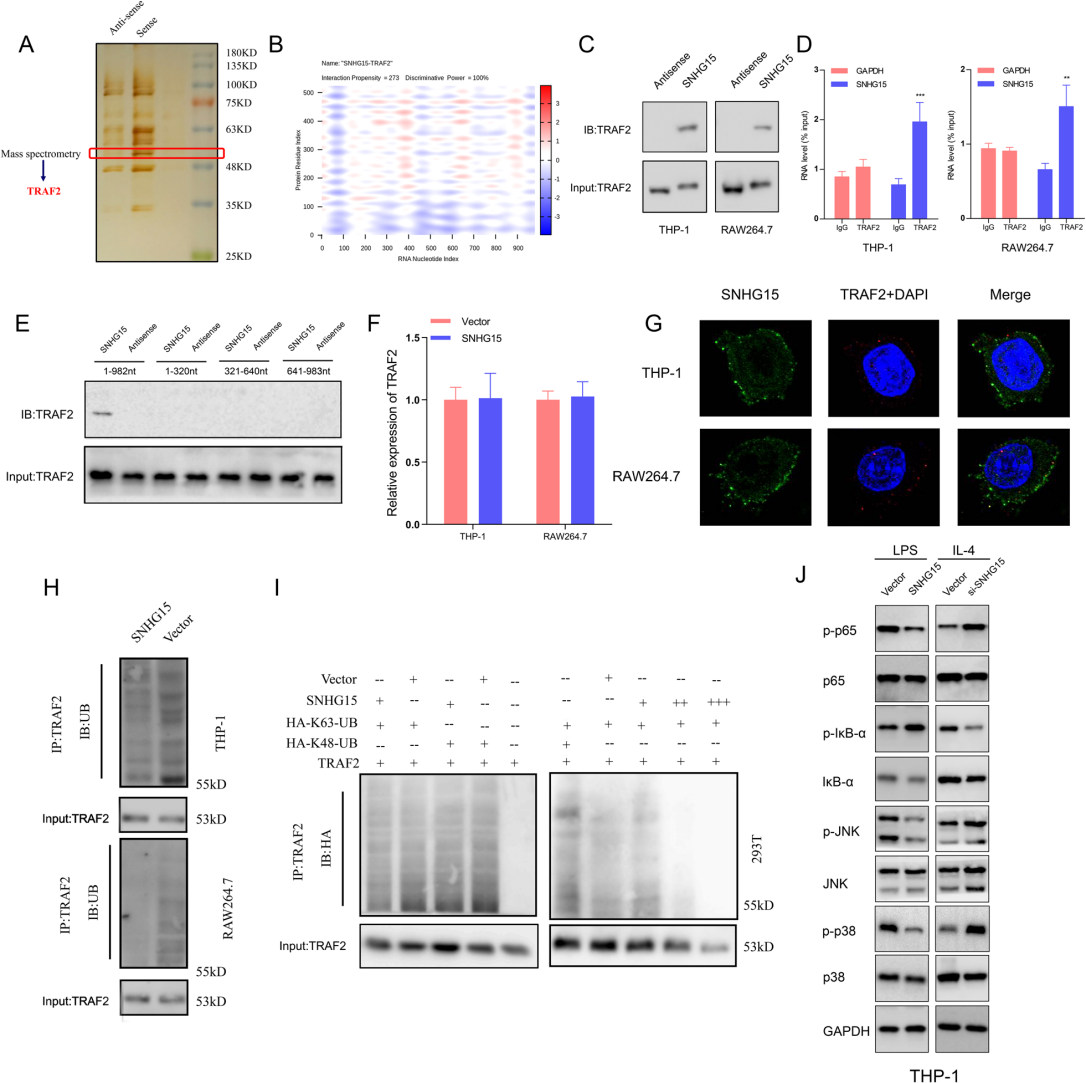
SNHG15 inhibits TRAF2 auto-ubiquitination. **A** Sliver-stained SDS-PAGE gels showing separated proteins in macrophages that were pulled down using a biotin-labeled SNHG15 probe. The bands in the highlighted regions were assessed by mass spectrometry, verifying TRAF2 as a protein specifically interacting with SNHG15. **B** The interaction of SNHG15 and TRAF2 was analyzed with the catRAPID database. **C** Western blot analysis of biotin-labeled SNHG15 probe-bound proteins in THP-1 and RAW264.7 cells using an anti-TRAF2 antibody. **D** RNA immunoprecipitation was performed with an anti-TRAF2 antibody to assess SNHG15 expression in macrophages by qRT-PCR. IgG was used as a control. **E** The interaction of TRAF2 with the full-length sequence or fragments of SNHG15 was assessed by RNA pull-down assays. **F** The effect of SNHG15 on the transcription level of TRAF2 was assessed by qRT-PCR. **G** The localization of SNHG15 in THP-1 and RAW264.7 cells was assessed by FISH. **H** Immunoblotting was performed to assess the ubiquitination of TRAF2 in THP-1 and RAW264.7 cells. **I** Immunoblot analysis of the ubiquitination of TRAF2 in HEK293T cells transfected to express TRAF2 and HA-tagged K48- or K63-linked ubiquitin. Right panel: The influence of different concentrations of SNHG15 on the ubiquitination of TRAF2. **J** Effects of forced expression or silencing of SNHG15 on MAPK and NF-κB in THP-1 and RAW264.7 cells were determined by western blotting. *** P < 0.001

Several cytoplasmic lncRNAs have been reported to interact with ubiquitination-associated proteins, thus regulating the process of ubiquitination and thereby controlling the activation of downstream pathways [19]. To explore potential SNHG15-interacting proteins in THP-1 cells, we performed a pull-down assay with biotinylated SNHG15 followed by mass spectrometry. TRAF2, which is a pivotal signaling protein in the activation of the canonical mitogen-activated protein kinase (MAPK) and nuclear factor kappa-B (NF-κB) pathways, was identified as a SNHG15-interacting protein (Fig. 5A). This result was verified by a module predicting RNA–protein interactions in the catRAPID database (Fig. 5B) as well as by independent immunoblotting in THP-1 and RAW264.7 cells (Fig. 5C), and the specificity of this interaction was further confirmed by a RIP assay (Fig. 5D). To identify the SNHG15-associated region of TRAF2, we established three pairs of biotinylated fragments of SNHG15 (1–320 nt, 321–640 nt, 641–983 nt) and used them to perform a pull-down assay with THP-1 cell lysates, revealing that only full-length SNHG15 bound to TRAF2 (Fig. 5E). To determine the influence of SNHG15 on the transcription level of TRAF2, we conducted qRT-PCR in THP-1 and RAW264.7 cells. The findings suggested that the transcription level of TRAF2 remained unchanged in LPS-induced macrophages (Fig. 5F). Moreover, confocal microscopy to assess SNHG15 and TRAF2 immunostaining indicated that SNHG15 colocalized with TRAF2 in the cytoplasm of IL-4-activated macrophages (Fig. 5G). These findings revealed that SNHG15 physically interacts with TRAF2 in macrophages.

TRAF2 is commonly recruited to receptor complexes upon activation of Toll-like receptor 4 (TLR4) signaling to initiate downstream signaling (MAPK and NF-κB), and auto-ubiquitination of TRAF2 is essential for signal transduction [20]. To determine whether the interaction of SNHG15 with TRAF2 affects its ubiquitination, we performed a Co-IP assay and found that ubiquitination of TRAF2 was significantly suppressed by SNHG15 in cultured LPS-induced macrophages (Fig. 5H). To further determine the type of ubiquitin on TRAF2, HEK293T cells were transfected with plasmids to express mutant forms of HA-tagged ubiquitin (K48- or K63-linked). The presence of SNHG15 diminished the level of K63-linked ubiquitin in a dose-dependent manner, but the level of K48-linked ubiquitin on TRAF2 was not changed (Fig. 5I). These results revealed that SNHG15 suppresses the activating K63-linked ubiquitination of TRAF2 but does not affect K48-linked ubiquitination. In addition, the LPS–TLR4 signaling cascade was impacted by the downstream signaling pathways of TRAF2, including the MAPK and NF-κB pathways, which were significantly affected by forced expression or silencing of SNHG15 (Fig. 5J). Hence, SNHG15 interacts with TRAF2 and interferes with auto-ubiquitination of TRAF2 and the related inflammatory responses.

### Expression of upstream molecules of SNHG15 in macrophages

**Fig. 6:**
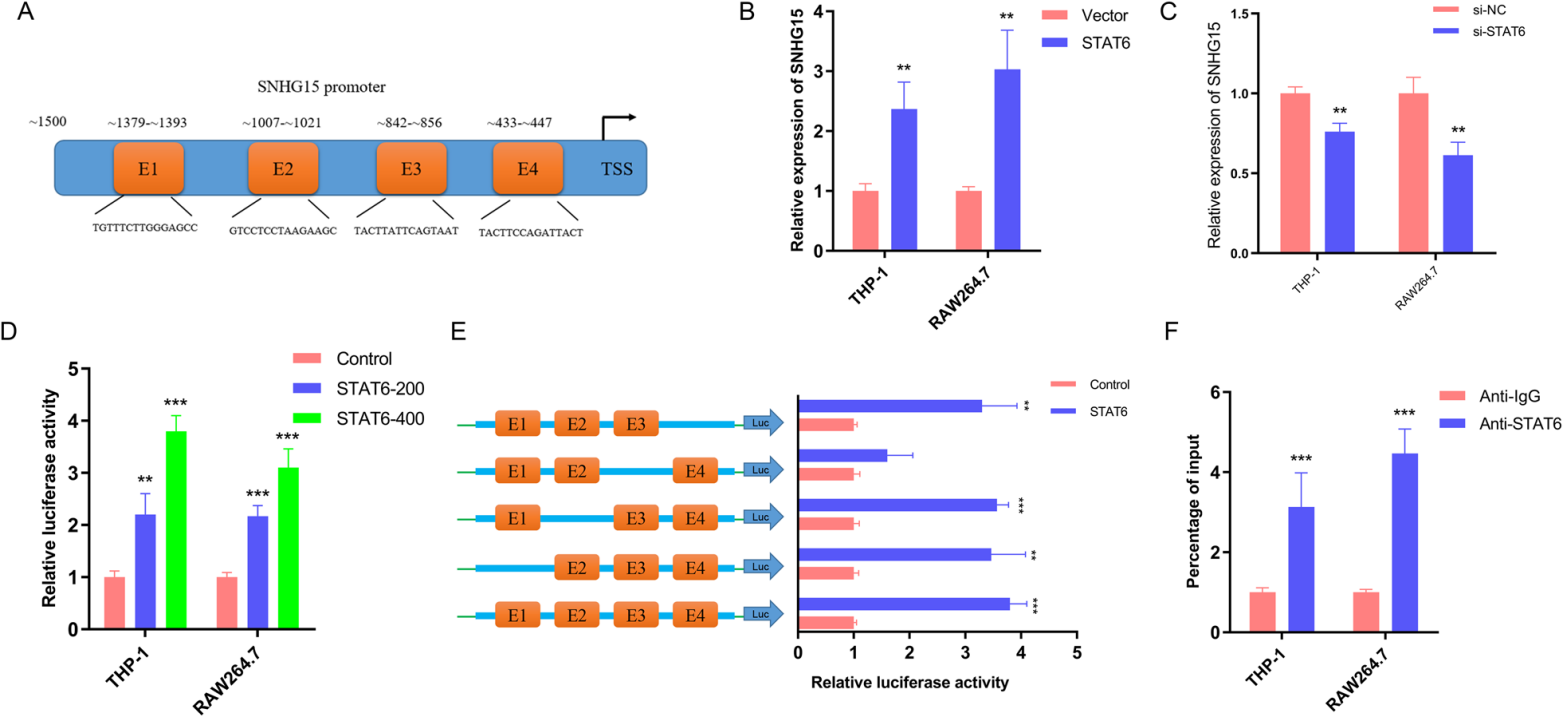
IL-4 induces SNHG15 expression in macrophages through JAK-STAT6 signaling. **A** The JASPAR CORE database was used to search for potential interacting regions in the SNHG15 promoter. **B** Effects of STAT6 overexpression on SNHG15 expression in macrophages. **C** Effects of STAT6 knockdown on SNHG15 expression in macrophages. **D** The interaction of STAT6 with the SNHG15 promoter was determined by luciferase reporter assays. **E** The STAT6 binding sites in the SNHG15 promoter were further determined by luciferase reporter assays. **F** STAT6 occupancy in the SNHG15 promoter region was analyzed by ChIP. ** P < 0.01, *** P < 0.001

To explore the mechanism underlying the abnormal expression of SNHG15 in activated macrophages, the JASPAR CORE database was used to search the potential interacting regions in the SNHG15 promoter [21]. Four putative STAT6 binding regions with relatively high scores—E1 (TGTTTCTTGGGAGCC, ~ 1379 to ~ 1393), E2 (GTCCTCCTAAGAAGC, ~ 1007 to ~ 1021), E3 (TACTTATTCAGTAAT, ~ 842 to ~ 856), and E4 (TACTTCCAGATTACT, ~ 433 to ~ 447—were selected (Fig. 6A). In view of the decreased STAT6 expression in LPS-induced macrophages [19], we observed that forced expression of STAT6 promoted SNHG15 expression in THP-1 and RAW264.7 cells activated by LPS (Fig. 6B), whereas an opposite effect was found after silencing of STAT6 in IL-4-induced macrophages (Fig. 6C). In line with these findings, STAT6 primarily bound to the E3 region in the SNHG15 promoter, as determined by luciferase reporter assays (Fig. 6D and E). Furthermore, ChIP assays performed at the E3 site in the SNHG15 promoter revealed that STAT6 directly interacted with the SNHG15 promoter region in cultured macrophages activated by IL-4 (Fig. 6F). These data showed that overexpression of SNHG15 in macrophages is partially due to STAT6 activation.

### SNHG15 is an anti-inflammatory molecule in mice

**Fig. 7:**
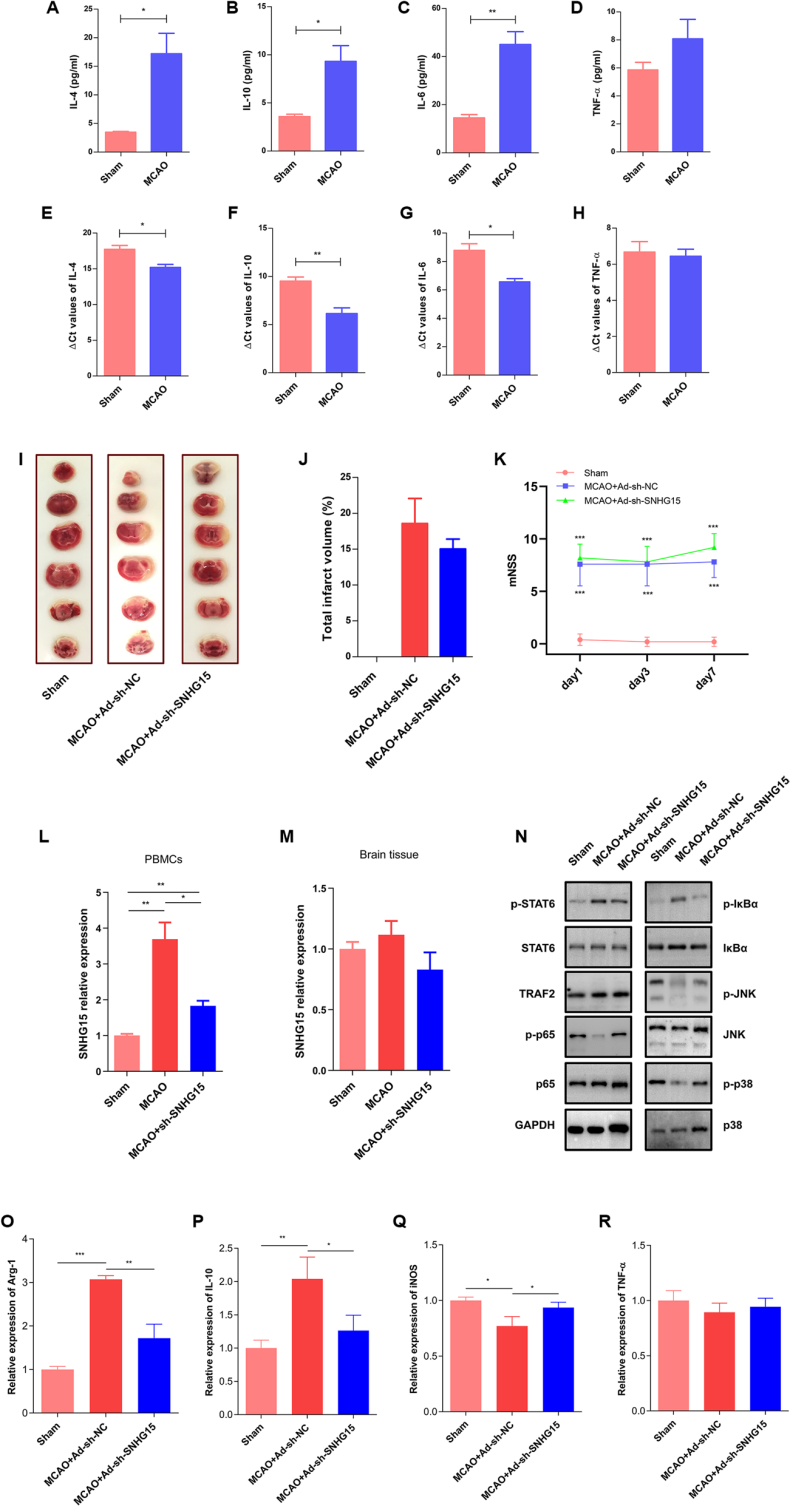
SNHG15 is an anti-inflammatory molecule in mice. The levels of IL-4 (**A**), IL-10 (**B**), IL-6 (**C**), and TNF-α (**D**) in plasma were measured by ELISA. Expression of IL-4 (**E**), IL-10 (**F**), IL-6 (**G**), and TNF-α (**H**) was detected by qRT-PCR in PBMCs. **I**, **J** Effects of SNHG15 knockdown on cerebral injury 3 days post-stroke. **K** Neurological function was assessed 1, 3 and 7 days after tMCAO. The modified neurological severity score (mNSS) was used by investigators blinded to the tMCAO and control groups to assess neurological deficits; the assessment included the tail suspension test, locomotor test, beam walking test and reflex test. Neurological function was graded on a scale of 0 to 14 points, and higher scores indicated more severe neurological deficits. SNHG15 expression in PBMCs (**L**) and brain tissues (**M**). **N** Effects of SNHG15 knockdown on the STAT6, TRAF2, NF-κB, and MAPK pathways 3 days post-stroke. **O**–**R** Effects of silencing SNHG15 on the expression of inflammatory factors 3 days post-stroke. * P < 0.05, ** P < 0.01, *** P < 0.001

Given the important roles of SNHG15 in the inflammatory response, we evaluated whether SNHG15 is involved in SIIS in the tMCAO mouse model. As expected, the levels of anti-inflammatory cytokines (IL-4 and IL-10) and a proinflammatory cytokine (IL-6) were found to be elevated in MCAO model mice by ELISA and qRT-PCR assays; however, the TNF-α level was not altered (Fig. 7A–H). To explore the underlying mechanism of SNHG15, we constructed an adenoviral vector targeting SNHG15 and injected this vector into mice through the tail vein. The results suggested that SNHG15 expression was increased in PBMCs after MCAO (Fig. 7L) but was not increased in brain tissues (Fig. 7M). Additionally, no marked difference in the total infarct volume or modified neurological severity score (mNSS) was observed between MCAO model mice in the Ad-sh-NC and Ad-sh-SNHG15 groups (Fig. 7I–K). To further determine the effects of SNHG15 expression on downstream signaling pathways, we performed immunoblotting to analyze related signaling proteins. The findings indicated that knockdown of SNHG15 facilitated activation of the MAPK and NF-κB pathways (Fig. 7N). Finally, the immune-inflammatory response was assessed through the detection of relevant markers in macrophages by qRT-PCR. We found that silencing SNHG15 accelerated macrophage polarization toward the M1 phenotype after MCAO (Fig. 7O–R).

## Discussion

Accumulating evidence indicates that lncRNAs are involved in central and peripheral immune systems after stroke [22, 23]. Stroke induces repression of the peripheral immune system, which contributes to the incidence of post-stroke infections [11]. In this study, we investigated lncRNA expression profiles in monocytes/macrophages after stroke and identified an lncRNA with stroke-induced upregulation (SNHG15). Activation of the IL-4–STAT6 pathway transcriptionally increased the expression of SNHG15, which was primarily localized in the cytoplasm. SNHG15 markedly impaired inflammatory responses through interference with TRAF2 auto-ubiquitination in the acute stage of ischemic stroke.

In recent years, numerous ncRNAs have been identified as biomarkers for stroke. Our group has explored miRNAs (miR-4443), lncRNAs (linc-DHFRL1-4, SNHG15 and linc-FAM98A-3) and circRNAs (circCDC14A, circFUNDC1 and circPDS5B) as potential diagnostic tools for AIS [17, 18, 24]. Here, SNHG15 was further evaluated in monocytes/macrophages from PBMCs identified as previously reported [17]. The present study first demonstrated that SNHG15 is involved in the process of SIIS, which increases the risk of SAI. SNHG15 was previously reported to be dysregulated in various types of cancers [25, 26]. Another study found that SNHG15 inhibited hyperglycemia-induced endothelial dysfunction by enhancing the ubiquitination of thioredoxin-interacting protein [27]. Interestingly, our study showed the role of SNHG15 in SIIS through abrogation of TRAF2 auto-ubiquitination, thereby blocking the MAPK and NF-κB signaling pathways.

A previous study reported that lncRNA-Mirt2 weakens the inflammatory response by inhibiting K63-linked ubiquitination of TRAF6 [19]. Another lncRNA, GAS5, was found to repress Th17 differentiation by potentiating TRAF6-associated ubiquitination of STAT3 [28]. TLR4/TRAF2 activation stimulated by LPS has been reported to initiate the related downstream signaling pathways, including the MAPK and NF-κB pathways, which results in the secretion of inflammatory cytokines [29–31]. Our findings showed an unexpected role for SNHG15 in mediating downstream TLR signaling pathways, such as the NF-κB and MAPK signaling cascades, by attenuating TRAF2 ubiquitination, but its transcription level was unchanged. We speculated that the interaction of SNHG15 with TRAF2 may hide its ubiquitination regions and thereby cause a decrease in the TRAF2 ubiquitination level. Thus, the mechanism underlying the roles of these proteins in macrophages requires further investigation.

Macrophage polarization, which is induced by LPS or IL-4, is a tightly regulated process that requires a series of signaling pathways [32]. M1 macrophages show proinflammatory responses that are essential for host defense, and M2 macrophages exhibit anti-inflammatory activities for the restoration of homeostasis [33]. In contrast to LPS stimulation, IL-4 stimulation resulted in a rapid rise in the SNHG15 level via the JAK-STAT6 signaling pathway, which was significant at 8 ng/mL. Silencing SNHG15 expression abrogated IL-4-induced M2 polarization with a marked increase in M2 markers, such as Arg-1, IL-10, iNOS, and TNF-α. Thus, SNHG15 facilitated M2 macrophage polarization and thereby aggravated immunosuppression after stroke.

Several limitations should be acknowledged in this study. Firstly, the small sample size limits the precise validation of human cytokines in AIS. Secondly, there are different approaches in the measurement of cytokine levels in human (flow cytometry) and mouse plasma samples (ELISA), which may result in the heterogeneity of findings.

## Conclusions

**Fig. 8:**
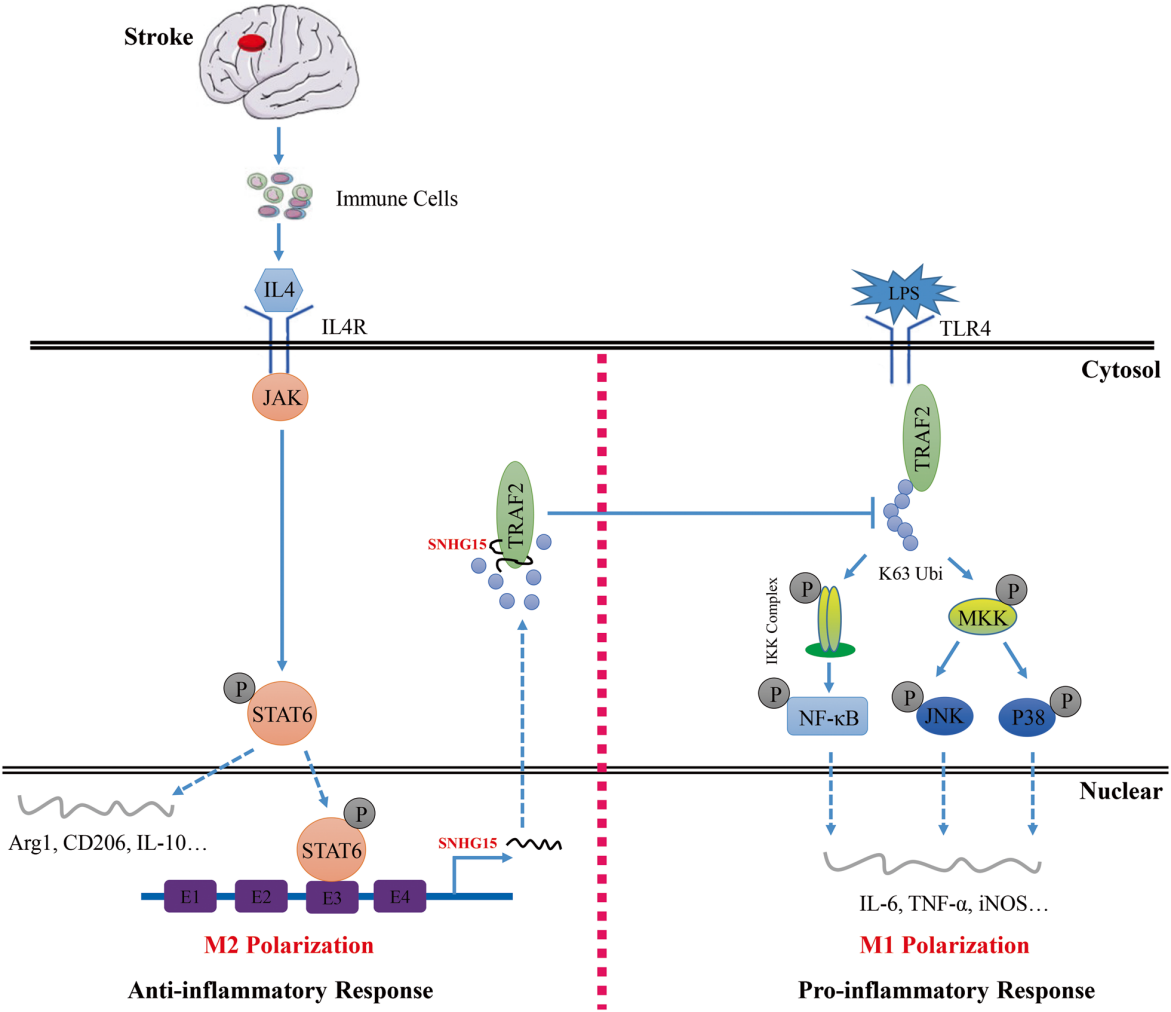
A schematic diagram of SNHG15-based signaling circuit in stroke-induced immunosuppression. Activation of IL-4–JAK-STAT6 signaling pathway contributes to the expression of SNHG15, and SNHG15 uniquely inhibits the K63-linked ubiquitination of TRAF2, thereby attenuating inflammatory responses and promoting M2 macrophage polarization after stroke

In summary, our present work provides novel insights into inflammatory responses involved in stroke-induced immunosuppression. SNHG15, which is an IL-4-induced lncRNA in macrophages, uniquely represses K63-linked ubiquitination of TRAF2 to promote M2 macrophage polarization and thereby attenuates inflammatory responses after stroke (Fig. 8).

## Supplementary Information

**Additional file 1.** Additional figures and tables.

## Data Availability

All data supporting our results are available from the corresponding authors upon reasonable request.

## Abbreviations

lncRNA: Long noncoding RNA
PBMC: Peripheral blood mononuclear cell
SIIS: Stroke-induced immunosuppression
SAI: Stroke-associated infection
TRAF2: TNF Receptor-associated factor 2
qRT-PCR: Quantitative real-time PCR
TNF-α: Tumor necrosis factor α
tMCAO: Transient middle cerebral artery occlusion
AIS: Acute ischemic stroke
JAK: Janus kinase
STAT6: Signal transducer and activator of transcription 6
RIP: RNA immunoprecipitation
Co-IP: Co-immunoprecipitation
STAT6: Signal transducer and activator of transcription 6
ANOVA: Analysis of variance
iNOS: Inducible nitric oxide synthase
FISH: Fluorescence in situ hybridization
TLR4: Toll-like receptor 4
MAPK: Mitogen-activated protein kinase
NF-κB: Nuclear factor kappa-B
mNSS: Modified neurological severity score
