# Supplementary material for: SNHG15 is a negative regulator of inflammation by mediating TRAF2 ubiquitination in stroke-induced immunosuppression
Source: J Neuroinflammation. 2022 Jan 3;19:1. doi: 10.1186/s12974-021-02372-z (PMC8722265; doi:10.1186/s12974-021-02372-z)
Supplement: Supplementary file 1 — Additional file 1. Additional figures and tables. [file 12974_2021_2372_MOESM1_ESM.docx]

**Supplementary methods**

***Construction of lentiviruses and plasmids***

To generate lentiviral constructs, the entire sequence of human or mouse SNHG15 was amplified and inserted into the SNHG15 lentiviral vector (pLenti-GIII-CMV-GFP-2A-Puro), and we also constructed plasmids for overexpression of human and mouse STAT6. A lentiviral vector that only contained the green fluorescent protein (GFP) sequence was considered the negative control (pLenti-EV). To construct the lentiviral vector expressing siRNA against SNHG15 (si-SNHG15), siRNA was designed and validated with the optimal knockdown efficiency and was then inserted into a lentiviral vector (pLenti-si-SNHG15). Additionally, a siRNA against STAT6 was constructed by RiboBio. The target sequence is listed in Supplementary Table 2. Lentiviral vectors were obtained from Hanbio Technology (Shanghai, China).

***Protein extraction and western blot analysis***

Cells were lysed in 50 μL of protein lysis buffer supplemented with 5 μL of phosphatase inhibitors, 0.5 μL of a protease inhibitor and 2.5 100 mM PMSF (KeyGEN BioTECH, Nanjing, China). Protein extraction and western blot analysis were performed as described in our previous study [32337817]. Briefly, after determination by a bicinchoninic acid (BCA) protein assay (Beyotime, Shanghai, China), total protein samples were resolved on 10% polyacrylamide gels and were then electroblotted onto polyvinylidene fluoride membranes (Merck Millipore, Billerica, MA, USA). Subsequently, the membranes were blocked with 5% nonfat milk (Sigma–Aldrich, St. Louis, MO, USA) and incubated with primary antibodies specific for NF-κB p65 (1:1000, Proteintech Group, Rosemont, USA, 10745-1-AP), phospho-NF-κB p65 (1:1000, CST, MA, USA, 3033S), IκB-α (1:1000, Proteintech Group, Rosemont, USA, 10268-1-AP), phospho-IκB-α (1:500, CST, MA, USA, 2859S), STAT6 (1:4000, Proteintech Group, Rosemont, USA, 66717-1-Ig), phospho-STAT (1:500, CST, MA, USA, 56554S), TRAF2 (1:2000, Abcam, Cambridge, US, ab126758), JNK (1:1000, CST, MA, USA, 9252S), phospho-JNK (1:1000, CST, MA, USA, 4668S), p38 MAPK (1:1000, Proteintech Group, Rosemont, USA, 14064-1-AP), phospho-p38 MAPK (1:1000, Cell Signaling Technology, MA, USA, 4511S), and GAPDH (1:2000, Engibody Biotechnology, Dover, USA, AT0010) overnight at 4 °C. Then the membranes were incubated with a horseradish peroxidase-conjugated goat anti-rabbit secondary antibody (Biosharp, Hefei, China, BL003A) and a goat anti-mouse secondary antibody (Biosharp, Hefei, China, BL001A) for 2 hours at room temperature. Meilunbio fg super sensitive ECL reagent (Meilunbio, Dalian, China) and a GE ImageQuant LAS4000 mini imaging system (GE, USA) were used for quantification of band intensities on the membranes.

***Mice***

All animal experimental procedures were conducted in accordance with the National Institutes of Health Guide for the Care and Use of Laboratory Animals and approved by the Institutional Animal Care and Use Committee at Nanjing Medical University. All animals were housed in a room with a constant temperature and humidity on a constant 12-hour light/12-hour dark cycle. Food and water were available *ad libitum*. Adult male C57BL/6J mice (18.0-20.0 g, 6-8 weeks old) were purchased from the Minhang Laboratory Animal Center of East China Normal University, and after adaptation for 1-2 weeks, the mice (24.0-26.0 g, 8-10 weeks old) were randomly assigned for animal experiments.

***Transient middle cerebral artery occlusion (tMCAO) model***

To establish the stroke model, male mice were anesthetized; anesthesia was induced with 3% sevoflurane (RWD Life Science Co., Shenzhen, China) and maintained with 1.0% -2.0% sevoflurane in 70% nitrous oxide and 30% oxygen delivered via a face mask from an animal anesthesia machine (RWD510, RWD Life Science Co., Shenzhen, China). A small midline incision was made in the neck skin overlying the trachea to carefully expose the right common carotid artery, external carotid artery and internal carotid artery. The common carotid artery and external carotid artery were ligated to obstruct the blood flow, and one silk suture was passed under the common carotid artery to fix the nylon filament. Then, a partial arteriotomy was performed at the distal end of the common carotid artery, and a 0.22-0.23 mm diameter silicone rubber-coated nylon filament (RWD Life Science Co., Shenzhen, China) was inserted through the incision to the internal carotid artery and advanced 9-110 mm along the carotid bifurcation to the middle cerebral artery. The rectal temperature was measured with a rectal probe and maintained at 37-38 °C with a heating pad (RWD Life Science Co., Shenzhen, China) during the surgery. After 60 min of occlusion, the filament was gently withdrawn and removed from the common carotid artery. The incision was sutured, and the mice were allowed to recover from anesthesia on a temperature-controlled heating pad. The mice in the sham-operated group underwent the same surgical procedure on the common and external carotid arteries except that no filament was inserted.

***Adenoviral vector construction and injection***

Recombinant adenovirus carrying the vector pAdenoG-siRNA-green fluorescent protein (GFP) targeting the lncRNA SNHG15 (Ad-sh-SNHG15) or the control vector (Ad-sh-NC) was produced and packaged at ABM (Applied Biological Materials Inc., Richmond, BC, Canada). Six siRNAs for mouse SNHG15 were designed by RiboBio (RiboBio Co., Guangzhou, China), and the siRNA with the optimal interference efficiency was chosen to construct the shRNA and was then cloned into adenoviral vectors (Hanbio Technology, Shanghai, China). The target sequence was as follows: GCAGUCUUUGUCCAUGAAA. Adenovirus (2.5×10^9^ pfu/mouse) and related adenovirus controls were injected into mice via the tail vein 7 days before the tMCAO model was established.

***Neurological deficit evaluation***

Neurological function was assessed 1, 3 and 7 days after tMCAO. The modified neurological severity score (mNSS) was used by investigators blinded to the tMCAO and control groups to assess the neurological deficits, as described elsewhere (20). Briefly, the tail suspension test, locomotor test, beam walking test and reflex test were included in assessment of the mNSS. Neurological function was graded on a scale of 0 to 14 points, and higher scores indicated more severe neurological deficits.

***Triphenyltetrazolium chloride (TTC) staining and infarct volume assessment***

The infarct volume was assessed 3 days after tMCAO in accordance with the time of neurological deficit evaluation. For TTC staining, the brain was removed rapidly and frozen at -20 °C for 5 min, and the brain was then coronally sectioned into 2 mm slices from the frontal tips in a brain matrix at -20 °C. The sections were incubated with 2% TTC (Sigma–Aldrich, St. Louis, MO, USA) at 37 °C for 10-15 min and were then fixed in a 4% paraformaldehyde solution overnight. The lesion volume (LV), contralateral hemisphere volume (CV), and ipsilateral hemisphere volume (IV) were quantified with ImageJ software (NIH, Bethesda, Maryland, USA) after images of the brain slices were digitized. The infarct volume was calculated as [LV- (IV-CV)]/CV×100% to correct for brain edema. The overall infarct volume was determined by summing the infarct volumes for all slices of each mouse brain.

***Mouse blood collection and PBMC preparation***

Mouse blood samples were obtained from the retro-orbital plexus by enucleation to rupture the ophthalmic artery. Peripheral blood samples (1-1.5 ml) were collected in K2 EDTA-containing tubes (Vacutainer, BD) and processed within 2 hours. After centrifugation at 1500 *rpm* for 10 minutes to remove plasma, blood samples were subjected to Ficoll density centrifugation (TBD science, Tianjin, China) to isolate PBMCs according to the manufacturer’s instructions.

**References**

1. Li Y, Chopp M, Chen J, Wang L, Gautam SC, Xu Y-X, et al. Intrastriatal Transplantation of Bone Marrow Nonhematopoietic Cells Improves Functional Recovery After Stroke in Adult Mice. Journal of Cerebral Blood Flow & Metabolism. 2000;20(9):1311-9.

**Supplementary Table 1. The sequences of qRT-PCR primers**

| Gene | Forward (5’-3’) | Reverse (5’-3’) |
| --- | --- | --- |
| Human linc-DHFRL1-4 | AAGCGTTCAAGCTCAACACC | TGTAGGCTTATGCGGAGGAG |
| Human linc-FAM98A-3 | CTCCGCACACTGGATGAGAA | TTCAGCTTCGCTGGAGAGGT |
| Human SNHG15 | TCTTGGCTGGCAGACCTGTA | CAGGAATGGTCAGGCAACAC |
| Human IL-6 | CAGACAGCCACTCACCTC | CTCAAACTCCAAAAGACCAG |
| Human TNF-α | TGTAGCAAACCCTCAAGC | GGACCTGGGAGTAGATGAG |
| Human IL-10 | GGAGAACCTGAAGACCCT | TGATGAAGATGTCAAACTCACT |
| Human IL-4 | AGAAGGACACTCGCTGCCTG | GGTTCCTGTCGAGCCGTTTC |
| Human Arg-1 | GGACCTGCCCTTTGCTGACATC | TCTTCTTGACTTCTGCCACCTTGC |
| Human iNOS | CCTTCAGTATCACAACCTCAGCA | TGGAGACTTCTTTCCCGTCTC |
| Human TRAF2 | GACGTGAAGGCGCACCACGA | ACCGTCTCGAGGCAGCCGAT |
| Human GAPDH | GGGAGCCAAAAGGGTCATCA | TGATGGCATGGACTGTGGTC |
| Human U6 | CAGCACATATACTAAAATTGGAACG | ACGAATTTGCGTGTCATCC |
| Mouse SNHG15 | TCTTCCGTCATCAGGGCACAGG | TCCACCGCAGGCTGATCTCC |
| Mouse IL-6 | CTCTGAAGGACTCTGGCTTTG | GATGCTACCAAACTGGATATAATCAG |
| Mouse TNF-α | CATCTTCTCAAAATTCGAGTGACAA | TGGGAGTAGACAAGGTACAA |
| Mouse IL-10 | CAGGGATCTTAGCTAACGGAAA | GCTCAGTGAATAAATAGAATGGGAAC |
| Mouse IL-4 | TACCAGGAGCCATATCCACGGATG | TGTGGTGTTCTTCGTTGCTGTGAG |
| Mouse Arg-1 | GGCAAGGTGATGGAAGAGAC | AGGTGAATCGGCCTTTTCTT |
| Mouse iNOS | AGCAACTACTGCTGGTGGTG | TCTTCAGAGTCTGCCCATTG |
| Mouse GAPDH | CGACTTCAACAGCAACTCCCACTCTTCC | TGGGTGGTCCAGGGTTTCTTACTCCTT |

small nucleolar RNA host gene 15, SNHG15; interleukin, IL; qRT-PCR, quantitative real-time PCR; arginase 1, Arg-1; inducible NO synthase, iNOS; tumour necrosis factor, TNF; TNF receptor-associated factor 2, TRAF2.

**Supplementary Table 2. The sequences of probes**

| **FISH probe** | **Sequences** |
| --- | --- |
| SNHG15 | GGGTCCTGAGGCTGCGTCTGCGTGT |
| **si-RNA** |  |
| Human si-SNHG15 | GCAAGCCUUGGCACCUUAA |
| Mouse si-SNHG15 | GCAGUCUUUGUCCAUGAAA |
| **CHIRP probes** |  |
| Probe 1 | AACTTGCTCAATTAAGGTGC-/3bio/ |
| Probe 2 | ATTCAGGAGATACCAGAAGG-/3bio/ |
| Probe 3 | AAGACTGCTTGAACATGAGA-/3bio/ |
| Probe 4 | TATCTTCTCTCAGGTCAGGT-/3bio/ |
| Probe 5 | GTAGAGTTTAGGGTCCTGAG-/3bio/ |
| Probe 6 | GATGACTAGACTGCCGAAGA-/3bio/ |


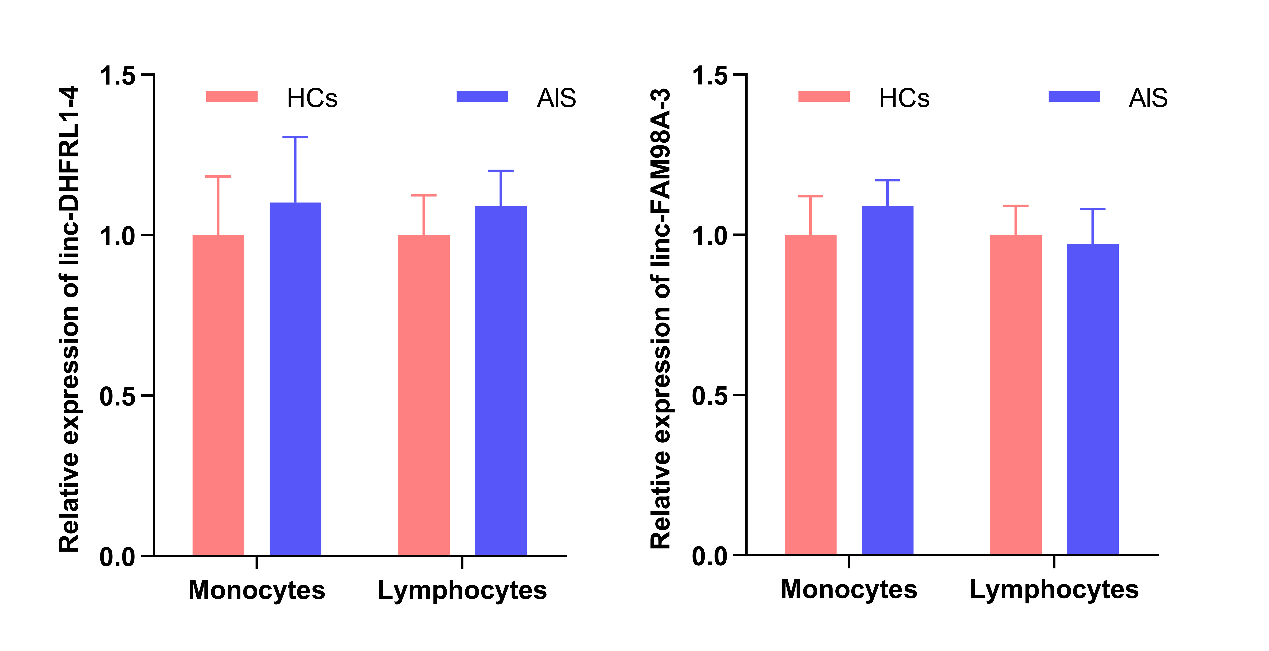


**Figure S1. Linc-DHFRL1-4 and linc-FAM98A-3 expression in the subpopulations of PBMCs from AIS.**


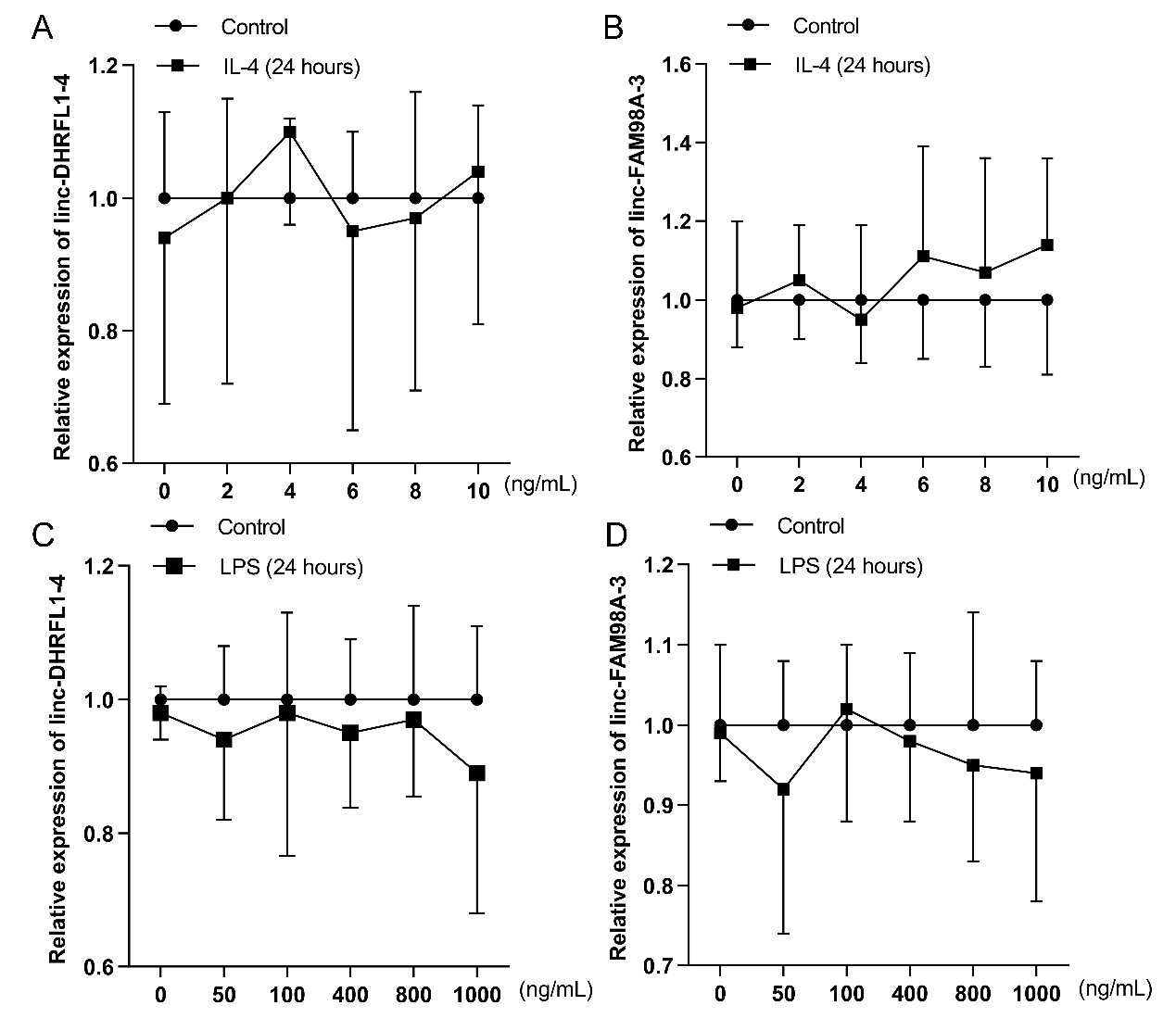


**Figure S2. The expression of linc-DHFRL1-4 and linc-FAM98A-3 in IL-4-induced (A-B) or LPS-induced (C-D) monocytes isolated from PBMCs.**


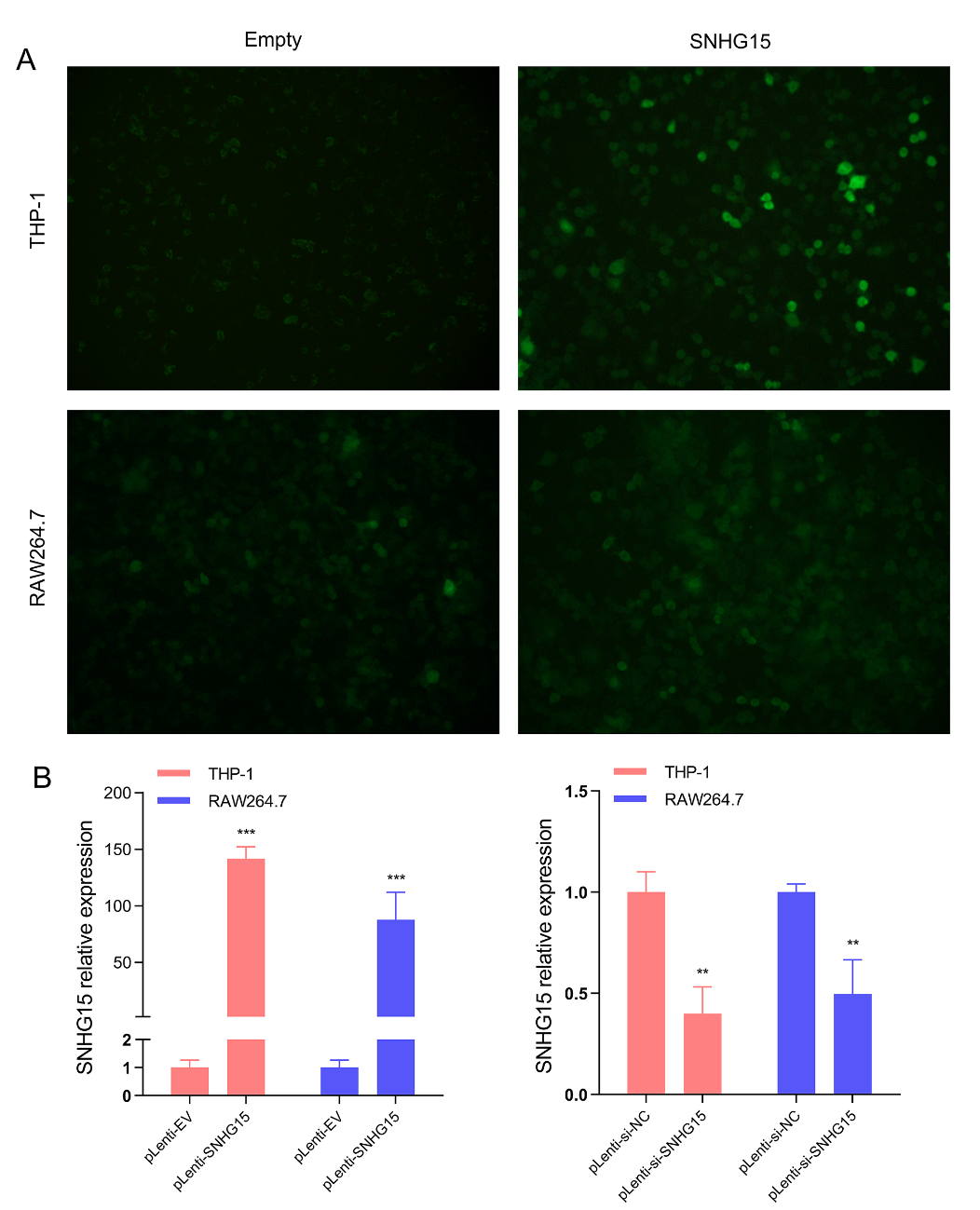


**Figure S3.** (A) The entire sequences of human or mouse SNHG15 were amplified and cloned into the SNHG15 lentiviral vector (pLenti-GIII-CMV-GFP-2A-Puro), which was transduced into THP-1 and RAW264.7 cells. Empty vector was also cloned into the lentiviral vector as a control. (B) The transfection efficiency of SNHG15 was verified by qRT–PCR. ^**^*P*<0.01, ^***^*P*<0.001.
